# Atrial Fibrillation Screening in Those with Rheumatic Heart Disease: A Narrative Policy Content Review

**DOI:** 10.5334/gh.1552

**Published:** 2026-04-28

**Authors:** Elizabeth Paratz, Xinying Zhou, Caio A. M. Tavares, Priyansh Shah, Julian Hoevelmann, Menglu Ouyang, Diana H. P. Foo, Amitava Banerjee, Ben Freedman, James Marangou, Jessica J. Orchard

**Affiliations:** 1 St Vincent’s Institute for Medical Research, Melbourne, Australia; 2 Sydney School of Public Health, The University of Sydney, Sydney, Australia; 3 Academic Research Organization (ARO), Hospital Israelita Albert Einstein, São Paulo, Brazil; 4 Instituto do Coracao (InCor), Hospital das Clinicas HCFMUSP, Faculdade de Medicina, Universidade de Sao Paulo, São Paulo, Brazil; 5 Department of Medicine, Albert Einstein College of Medicine/Jacobi Hospital, New York, United States; 6 Cape Heart Institute, Faculty of Health Sciences, University of Cape Town, Cape Town, South Africa; 7 Department of Internal Medicine III –Cardiology, Angiology, and Intensive Care Medicine, Saarland University Hospital, Saarland University, Homburg, Germany; 8 The George Institute for Global Health, Faculty of Medicine, UNSW, Sydney, Australia; 9 Clinical Research Center, Institute for Clinical Research, Sarawak General Hospital, Kuching, Malaysia; 10 Institute of Health Informatics, University College London, London, United Kingdom; 11 Department of Cardiology, University College London Hospitals NHS Trust, London, United Kingdom; 12 Heart Research Institute, Charles Perkins Centre, the University of Sydney, Sydney, Australia; 13 Menzies School of Health Research, Charles Darwin University, Darwin, Australia; 14 Department of Cardiology, Royal Perth Hospital, Perth, Australia

**Keywords:** rheumatic heart disease, atrial fibrillation, advocacy, screening, prevention

## Abstract

**Background:**

Rheumatic heart disease (RHD) and atrial fibrillation (AF) are major global health burdens. AF affects up to one-third of individuals with RHD, yet clinical guidelines lack recommendations for AF screening in this population.

**Aim:**

To identify and review current guidelines/policies for AF and RHD to assess whether recommendations are made for AF screening in patients with RHD, thereby identifying any policy gaps.

**Methods:**

A narrative policy content review was conducted. This included: guidelines, consensus statements, position statements and policy documents regarding AF or RHD. Documents were identified through searches in MEDLINE, national/international cardiovascular society websites and citation tracking. A narrative synthesis approach was used to review content.

**Results:**

A total of 29 documents were identified (16 focussed on AF; 13 on RHD). Of the 16 AF-focussed documents, 13 recommended AF screening in the general population in high-risk groups, based on age and comorbidities. Only one, the 2021 Asia Pacific Heart Rhythm Society (APHRS) AF practice guidance, explicitly recommended AF screening in higher-risk patients with RHD. The 13 RHD-focussed documents acknowledged AF as a frequent complication, particularly in advanced valvular disease, but none specifically recommended screening.

**Conclusion:**

This review highlights a clear gap in current guidelines and policies for AF screening in those with RHD. Although AF is a common complication of RHD, only one policy document was identified that provided specific recommendations for AF screening in patients with RHD. Further work is needed to inform and develop appropriate AF screening guidelines tailored to RHD populations in different settings.

## Introduction

Rheumatic heart disease (RHD) refers to chronic valvular damage resulting from repeated or severe episodes of acute rheumatic fever (ARF), an autoimmune response to group A streptococcal (GAS) infection, typically beginning in childhood. Despite being preventable through early diagnosis and effective secondary prophylaxis, RHD continues to impose a significant burden on global cardiovascular health, primarily due to deficiencies in early detection and long-term management.

It is estimated that around 30 million people live with RHD worldwide, with more than 300,000 deaths annually (1,2). The disease disproportionately affects low- and middle-income countries (LMICs), where 80% of cases occur, and vulnerable groups in high-income countries. In Africa, the estimated prevalence is 25.5 per 1000 (or 2550 per 100,000) using World Heart Federation (WHF) criteria (3). In Australia, the overall prevalence of RHD diagnoses is 69 per 100,000, however, the figures are up to 45 times higher in Indigenous Australians in northern Australia: in the Northern Territory, the prevalence of RHD in First Nations people is 3171 per 100,000 (4).

**Figure 1:**
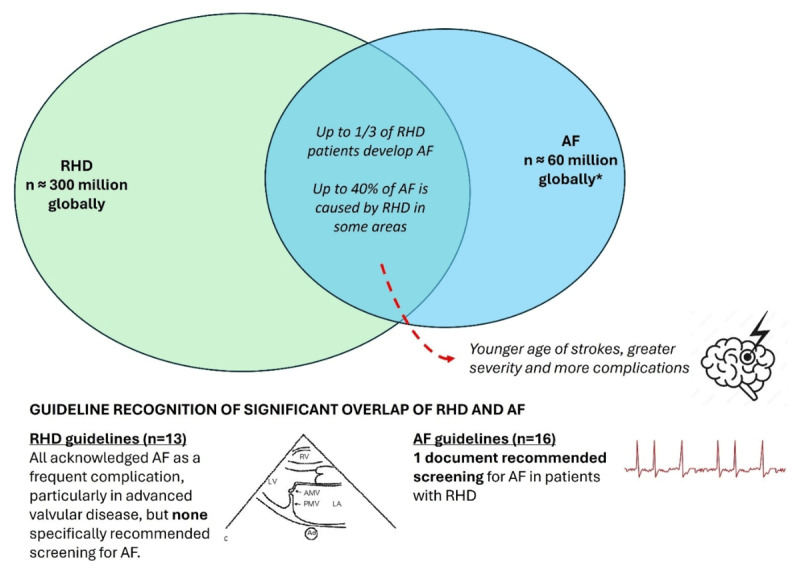
Despite the significant overlap of AF and RHD, only one guideline advocated for routine screening to occur. *Estimated AF numbers are likely inaccurate due to non-identification in people living with RHD – in systematically examined RHD populations, prevalence is up to 1 in 3, which would already create an estimate of up to 100 million cases globally. AF, atrial fibrillation; RHD, rheumatic heart disease.

Atrial fibrillation (AF) is the most common arrhythmia and is associated with a 5-fold increase in stroke risk as well as increased rates of mortality, heart failure and dementia (5,6,7). AF prevalence globally is estimated at 59 million people (8) (Figure 1). AF is strongly associated with age, and about 1.4% of people aged ≥65 years have undiagnosed AF, often without any symptoms (9). Undetected AF accounts for nearly 15% of ischemic strokes (7). Early identification and treatment with oral anticoagulants (OAC) for those at high risk can reduce stroke risk by 64% (10). Therefore, opportunistic screening is recommended by numerous guidelines for higher-risk groups, often defined by age. For example, the 2024 European Society of Cardiology (ESC) guidelines include a Class I Level C (‘recommended’, based on expert consensus) recommendation for ‘routine heart rhythm assessment’ of all people aged ≥65 years for earlier detection of AF. The ESC guidelines also include a Class IIa Level B (‘consider’, data from single randomised trial or large non-randomised studies) statement to consider population-based screening using prolonged monitoring for those aged ≥75 years or with additional risk factors (11). It is important to note that there are still gaps in the evidence for clinical outcomes of those screened for AF, and the ongoing SAFER international multicentre cluster randomised controlled trial will provide important data as to whether early detection of AF decreases stroke risk (12).

AF is a frequent and serious complication in patients with RHD. Up to one-third of RHD patients develop AF, and the incidence triples every 5 years after RHD diagnosis (13,14). However, there are still evidence gaps about risk factors for AF in those with RHD (15). Registry data show RHD causes 20%–40% of all AF in specific districts (15,16). RHD-related AF is more likely to occur at a young age and has a much higher stroke risk: up to 17-fold greater than non-valvular AF. Given that AF is more common and serious in those with RHD, there have been calls for guidelines on screening for early detection of AF and improved stroke prevention treatment in this group (14).

However, there are many gaps in the relevant guidelines and consensus documents. RHD remains under-recognised in most broad non-RHD focussed national and international policy frameworks (13). While regional guidelines from Europe, the Asia-Pacific and Australia endorse opportunistic or systematic AF screening in older adults or high-risk groups (7,17,18), few offer tailored recommendations for individuals with RHD, even though they are one of the highest risk groups for developing AF. This study aimed to identify and review current guidelines and policies for AF and RHD to assess the extent to which these documents address AF screening in RHD populations, thereby defining any policy gaps.

## Methods

This study used a narrative policy content analysis to quantify the degree to which current policy documents and guidelines do or do not include screening AF recommendations for RHD patients.

### Search strategy

A comprehensive literature search was conducted in May 2025 across multiple data sources to identify relevant national/international clinical guidelines and policy documents addressing AF screening in patients with RHD. Major biomedical databases (PubMed, MEDLINE and Scopus), were searched to capture peer-reviewed literature. In parallel, manual searches of other sources were conducted by reviewing the official websites of leading international and regional cardiovascular organisations, such as the World Health Organization (WHO), World Heart Federation (WHF), ESC, Asia Pacific Heart Rhythm Society (APHRS) and national cardiac and public health authorities (e.g., Communicable Diseases Network, Australia). Reference lists of key publications were manually screened to identify additional relevant documents.

The search strategy employed a combination of controlled vocabulary (e.g., MeSH terms) and free-text keywords, using Boolean operators to maximise search sensitivity. Core search terms included: ‘atrial fibrillation’, ‘rheumatic heart disease’, ‘screening’, ‘policy’, ‘guideline’, ‘recommendation’, ‘consensus statement’ and ‘position paper.’ The search targeted both AF-specific and RHD-specific guidelines, as well as broader cardiovascular policy documents that might contain relevant screening recommendations for high-risk populations.

### Inclusion and exclusion criteria

Documents were included if they met the following criteria:

1. They were official policy documents, guidelines, position papers or consensus statements published or endorsed by professional societies, expert groups or government bodies;
2. The content explicitly addressed AF or RHD, or both;
3. Were relatively recent (defined as published after 2010); and
4. The document was the most current version available when the search was conducted.

Exclusion criteria were: outdated versions, duplicate publications, non-official editorials, news articles, non-English language documents and commentaries lacking formal endorsement.

## Data extraction and synthesis

For each included document, data were extracted on the issuing organisation, publication year, document type, primary disease focus and the presence or absence of RHD-specific AF screening recommendations. A narrative synthesis approach was used to compare policy content, summarise areas of consensus and divergence and identify gaps in current guideline recommendations that may warrant further research or policy development.

## Results

**Figure 2:**
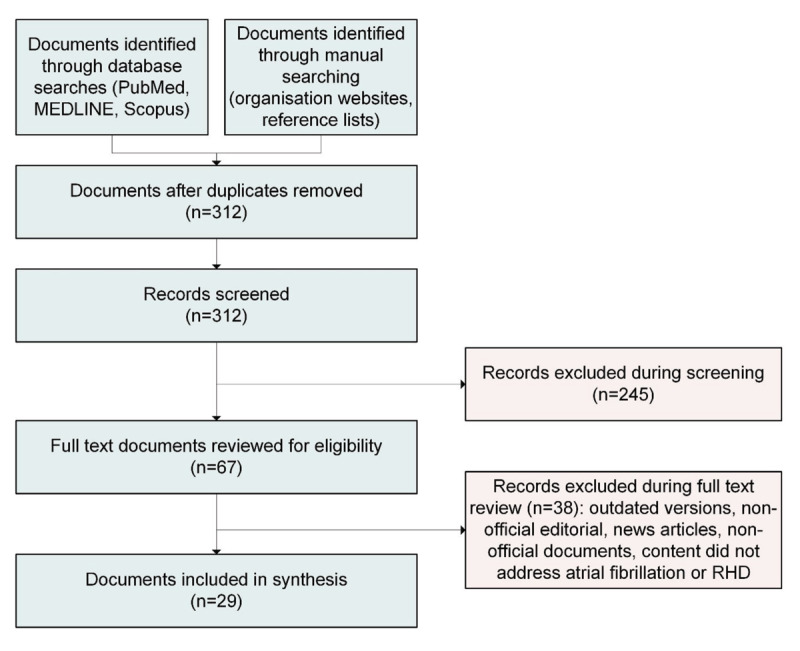
Flowchart of document identification and review process.

A total of 29 policy documents were included, comprising 13 guidelines primarily focussed on RHD and 16 focussed on AF (Figure 2). These documents were issued by a range of international, regional and national professional societies and health organisations and were published between 2012 and 2024. Of the 29 documents, only the 2021 practice guidance issued by the Asia Pacific Heart Rhythm Society (APHRS) provided explicit recommendations for AF screening in patients with RHD.

### AF-focussed guidelines: Higher-risk groups mainly based on age

Among the 16 AF-focussed policy and guideline documents reviewed, all but two supported some form of AF screening (systematic and/or opportunistic screening; Table 1), typically in well-defined higher-risk populations such as older adults or individuals with comorbidities or symptoms (19). For example, the 2024 ESC guidelines recommend opportunistic screening (e.g., pulse palpation or single lead ECG) for individuals aged ≥65 years and consideration of systematic ECG screening for those aged ≥75 years or at elevated stroke risk (11). Rather than relying on age, the UK National Institute for Health and Care Excellence (NICE) recommends pulse palpation ‘if there is a suspicion of AF’, for example, in people presenting with breathlessness, palpitations, syncope, dizziness, chest pain, stroke or transient ischaemic attack (20).

**Table 1:** Key characteristics of included AF-focussed guidelines, policies and documents.


TITLE AND TYPE	DOCUMENT, YEAR	LMIC OR HIC SETTING?	ORGANISATION(S)	MENTIONS RHD?	INCLUDES RECOMMENDATIONS?	

					AF SCREENING	AF SCREENING IN THOSE WITH RHD

**1. 2012 RCPE UK Expert consensus**	Stott et al., 2012 (22)	HIC	Royal College of Physicians of Edinburgh (RCPE), UK	Yes	Yes	No

**2. 2015 HIQA Health Technology Assessment**	HIQA, 2015 (23)	HIC	Health Information and Quality Authority, Ireland	No	Yes	No

**3. 2015 Brazilian Guidelines for AF**	Magalhaes et al., 2016 (24)	LMIC	Brazilian Society of Cardiology	No	No	No

**4. 2016 EPCCS Consensus guidance**	Hobbs et al., 2016 (25)	HIC	European Primary Care Cardiovascular Society (RPCCS)	Yes	Yes	No

**5. 2017 EHRA Expert consensus**	Mairesse et al., 2017 (26)	HIC	European Heart Rhythm Association (EHRA), endorsed by Heart Rhythm Society (HRS), Asia Pacific Heart Rhythm Society (APHRS), Sociedad Latinoamericana de Estimulacion Cardiaca y Electrofisiologia (SOLAECE).	No	Yes	No

**6. 2017 AF-SCREEN Expert consensus**	Freedman et al., 2017 (7)	n/a – INT	AF-SCREEN International Collaboration	Yes	Yes	No

**7. 2018 Aus/NZ Guideline**	Brieger et al., 2018 (17)	HIC	National Heart Foundation of Australia & Cardiac Society of Australia and New Zealand (CSANZ)	Yes	Yes	No

**8. 2018 Korean Guideline**	Joung et al., 2018 (27)	HIC	Korean Heart Rhythm Society (KHRS)	No	Yes	No

**9. 2020 WHF Roadmap**	Freedman et al., 2021 (28)	n/a – INT	World Heart Federation (WHF)	Yes	Yes	No

**10. 2020 CCS/CHRS Guideline**	Andrade et al., 2020 (29)	HIC	Canadian Cardiovascular Society/Canadian Heart Rhythm Society	Yes	Yes	No

**11. 2021 APHRS Practice guidance**	Chan et al., 2021 (15)	n/a – INT	Asia Pacific Heart Rhythm Society	Yes	Yes	Yes

**12. 2021 NICE Guideline**	NICE, 2021 (20)	HIC	National Institute for Health and Care Excellence (NICE), UK	No	Yes	No

**13. 2022 USPSTF Recommendation statement**	Davidson et al., 2022 (30)	HIC	US Preventive Services Task Force (USPSTF)	No	No	No

**14. 2023 ACC/AHA/ACCP/HRS Guideline**	Joglar et al., 2023 (21)	HIC	American College of Cardiology (ACC), American Heart Association (AHA), American College of Chest Physicians (ACCP), Heart Rhythm Society (HRS)	Yes	No	No

**15. 2024 Chinese Guideline**	Ma et al., 2024 (31)	LMIC	Chinese Society of Cardiology (CSC), Chinese Medical Doctor Association (CMDA)	No	Yes	No

**16. 2024 ESC Guideline**	Van Gelder et al., 2024 (11)	HIC	European Society of Cardiology (ESC)	Yes	Yes	No


HIC, high-income country; LMIC, low- and middle-income country; n/a – INT, not applicable – international paper.

The two documents that did not recommend AF screening in the general population were the US Preventive Services Task Force (USPSTF) and the 2023 American College of Cardiology (ACC)/American Heart Association (AHA) guideline. The USPSTF found that current evidence is ‘insufficient to assess the balance of harms of screening for AF’ (‘I statement’), and the ACC/AHA concluded that there was insufficient evidence of benefit from screening (21).

Nine of the 16 documents mention RHD, but typically only in the context of broader discussions on valvular heart disease or anticoagulation therapy. In most cases, RHD is used to help define ‘valvular AF’, which carries a distinct thromboembolic risk profile and influences anticoagulation strategy. However, these documents do not recognise RHD patients as a subgroup warranting specific AF screening strategies. The notable exception is the 2021 APHRS consensus guidance, which was developed with explicit consideration of regions where RHD remains endemic. It is the only AF practice guidance to directly recommend screening in patients with RHD.

### RHD-focussed guidelines: AF recognised as a complication

Among the 13 RHD-focussed guideline and policy documents reviewed, AF was most commonly discussed as a complication of advanced valvular disease (Table 2). While 10 of the documents explicitly mentioned AF in the context of RHD, such references were typically brief and embedded within broader discussions of chronic disease complications or anticoagulation. Notably, none of the guidelines reviewed recommend screening for AF in patients with RHD, even though several recognise the elevated thromboembolic risk associated with valvular AF.

**Table 2:** Key characteristics of included RHD-focussed guidelines.


TITLE & TYPE	DOCUMENT, YEAR	LMIC OR HIC SETTING?	EVALUATION OF AF RISK IN RHD	RECOMMENDATIONS ON AF SCREENING IN RHD

**1. 2003 Western Cape Guideline**	Western Cape Government, South Africa, 2003 (37)	HIC	No	No

**2. 2012 Kimberley RHD Guideline**	Kimberley Aboriginal Medical Services Council & WA Country Health Service, Australia, 2012 (40)	HIC	Yes	No

**3. 2015 India handbook**	Ministry of Health and Family Welfare, India, 2015 (38)	LMIC	No	No

**4. 2018 National Guidelines for Public Health Units**	Communicable Diseases Network, Australia, 2018 (41)	HIC	Yes	No

**5. 2017 Fiji Guideline**	Ministry of Health and Medical Services, Fiji, 2017 (33)	HIC	Yes	No

**6. 2018 Kenya Guideline**	Ministry of Health, Kenya, 2018 (34)	LMIC	Yes	No

**7. 2018 WHO Report**	WHO, 2018 (39)	n/a – INT	No	No

**8. 2020 AHA Scientific Statement**	Kumar et al., 2020 (42)	HIC	Yes	No

**9. 2020 JCS/JSCS/JATS/JSVS Valvular Heart Disease Guideline**	Izumi et al., 2020 (43)	HIC	Yes	No

**10. 2024 WHO Guideline**	WHO, 2024 (2)	n/a – INT	Yes	No

**11. 2024 New Zealand Guideline**	Health New Zealand, 2024 (32)	HIC	Yes	No

**12. 2024 Sudan Guideline**	Sulafa et al., 2024 (35)	LMIC	Yes	No

**13. 2025 Australian Guideline**	RHD Australia, 2025 (36)	HIC	Yes	No


AHA, American Heart Association; HIC, high-income country; NSW, New South Wales; JCS, Japanese Circulation Society; JSCS, Japanese Society of Cardiac Surgery; JATS, Japanese Association for Thoracic Surgery; JSVS, Japanese Society for Vascular Surgery; LMIC, low- and middle-income country; n/a – INT, not applicable – international paper; RHD, rheumatic heart disease; WHO, World Health Organisation; WA, Western Australia.

In the 2024 WHO guideline, AF is listed among the major complications of symptomatic chronic RHD, alongside heart failure, stroke and endocarditis. The document advises that these conditions ‘should be managed using standard protocols, including anticoagulation where indicated (e.g., AF, mechanical valve)’, but offers no additional detail on detection or timing of intervention (2). Similarly, the New Zealand 2024 guideline identifies AF as one of several complications that may prompt the diagnosis of RHD in adulthood and includes AF in its management considerations for long-term follow-up and pregnancy, though without specifying detection strategies or thresholds for intervention (32).

Some national and regional guidelines from low- and middle-income settings, including Fiji (33), Kenya (34) and Sudan (35), recommend anticoagulation for patients with known AF and RHD. For instance, the Fiji guideline recommends long-term anticoagulation in ‘all patients with AF and valvular heart disease’, referring clinicians to cardiovascular disease guidelines for further AF management (33). The Sudan guideline lists AF as a high-risk condition in RHD patients that warrants intensified anticoagulation, grouping it with mitral stenosis, prior thromboembolism and reduced ejection fraction (35).

Among the highest resource environments, the most comprehensive set of recommendations on managing AF in RHD is available in Australia’s 2025 national RHD guideline (36). It includes specific indications for anticoagulation in patients with AF and rheumatic mitral stenosis, references the INVICTUS trial results in guiding agent selection, and discusses anticoagulation in patients in sinus rhythm with enlarged left atria or prior embolic events. However, despite this depth, the Australian guideline does not advocate for routine or opportunistic screening for AF in RHD populations.

In contrast, several older documents, such as the Western Cape (2003) and India’s 2015 handbook, do not mention AF at all (37,38). The 2018 WHO RHD policy report similarly omits AF, focussing instead on primary and secondary prevention of acute rheumatic fever and structural heart disease (39).

Across all RHD focussed-documents, discussion of AF is largely confined to treatment pathways initiated after clinical detection. There is no mention of asymptomatic or subclinical AF, nor guidance on when or how to assess for arrhythmias during routine RHD follow-up. While AF is increasingly recognised in newer guidelines as an important driver of morbidity – particularly through its association with stroke and heart failure – none of the RHD documents specifically incorporate AF screening recommendations for those with RHD.

## Discussion

This review of AF and RHD policies and guidelines shows that only one document includes specific recommendations for AF screening in those with RHD. These findings reveal an important policy gap, given the high risk of premature AF and stroke for RHD patients. While AF guidelines typically support screening in higher-risk groups, this is most commonly defined by age. For example, in Australia, the National Heart Foundation/Cardiac Society of Australia and New Zealand guidelines recommend opportunistic screening of people aged ≥65 years in the clinic or community (17). Conversely, while RHD guidelines acknowledge AF as a frequent and serious complication, they fail to include specific recommendations for early detection or systematic identification. Despite the lack of evidence for AF screening effectiveness in this population, guidelines do not explicitly acknowledge this evidence gap or offer expert consensus recommendations.

### AF screening recommendations in those with RHD

The only document that includes specific AF screening recommendations for those with RHD is the APHRS 2021 AF screening practice guidance (15). One of the most important contributions of the APHRS practice guidance is its explicit acknowledgement of RHD as one of the major causes of AF in LMICs. While the incidence of RHD has sharply declined in Western populations, it remains endemic in much of the Asia-Pacific and among Indigenous populations in Australia and New Zealand. Approximately 73% of global RHD cases are concentrated in five countries: India, China, Pakistan, Indonesia and the Democratic Republic of Congo (44). AF linked to RHD tends to occur at younger ages and carries a significantly elevated risk of thromboembolic events, particularly stroke. The APHRS document directly addresses this, offering the first structured, regionally contextualised policy framework for screening in this high-risk population.

To support implementation across diverse healthcare systems, the APHRS document has a pragmatic, three-tiered recommendation model according to national health resources. The levels of recommendation are: Level 1 (applicable to all countries), Level 2 (most countries) and Level 3 (some countries with higher resources). Within this framework, the guidance defines clear clinical indicators for prioritising RHD patients for screening. AF screening is recommended for RHD patients aged >50 years (all countries) and higher-risk RHD patients (most countries). ‘Higher risk’ RHD patients include those with NYHA class II or greater functional impairment, as well as those with echocardiographic markers of disease severity, including left atrial enlargement (>4.0 cm), mitral valve area <1.0 cm², mitral calcification, or a mitral valve gradient >10 mmHg. Such stratification provides operationally feasible and clinically relevant means of selecting RHD patients at the highest risk of developing AF. The guidelines suggest numerous options for screening methods, including pulse palpation, handheld single-lead ECGs, automated blood pressure monitors with software for detecting AF, and smartphone applications based on photoplethysmography (PPG). Diagnosis requires ECG verification (single or 12-lead), and a pathway to management and treatment with anticoagulation for those at high stroke risk.

There are a number of theoretical benefits of recommending AF screening for patients with RHD. Patients with RHD are more likely to develop AF at a younger age and with a higher risk (16). They are also more likely to present late and are frequently from LMICs, where RHD is more prevalent (14). Screening benefits in this group could include improved clinical outcomes from early detection and treatment and improved resource allocation and reduced health system burden from stroke prevention. There is also an important health equity argument in favour of improved screening and prevention in this patient population.

### Possible reasons for the lack of prioritising RHD

RHD does not receive a sufficiently high level of global priority. A recent review identified several reasons for this: (1) leadership and governance: RHD mainly affects low income groups in dispersed regions, making coordination of efforts more complicated; (2) solution definition: lack of agreement as to how best to address RHD; and (3) positioning: the perception of RHD as being mainly a problem for LMICs and ambiguity of its status as a noncommunicable disease have impeded action by policy makers (45). Another paper by Belay and Aliyu states bluntly that RHD is ‘missing from the global health agenda’ (46). The authors highlight a number of reasons, including underestimated morbidity, mortality and economic burden, lack of awareness and lack of sustainable investment.

In the context of AF screening policies, one other reason for a lack of inclusion of RHD is gaps in the evidence. While the APRHS practice guidance notes evidence of some predictors of AF in RHD [e.g., age >50 years (47), left atrial diameter >4.0 cm on echocardiogram (48), mitral valve calcification (49) and severity of mitral valve stenosis (50)], it also points out that more evidence is needed about risk factors in this population (15). A recent meta-analysis showed that the main factors associated with AF in those with RHD include age, mitral valve disease, especially with associated tricuspid involvement, left atrium enlargement, right atrium pressure and systolic pulmonary arterial pressure (14). The authors also noted that the development of a predictive tool to help identify patients at the highest risk for developing AF would be helpful in the RHD population (14).

### Health system impact and implications

In patients with RHD, particularly those with rheumatic mitral stenosis, the presence of AF acts as a profound risk multiplier, increasing the risk of stroke by up to 17-fold (51). The clinical benefit of early intervention is exceptionally high. The number needed to treat (NNT) to prevent one major stroke over a single year is estimated at only 12 to 15 patients (52,53). Beyond ischemic stroke, undetected AF in RHD rapidly accelerates the progression of severe heart failure and increases the risk of systemic embolization, frequently precipitating life-threatening cardiovascular crises. Furthermore, RHD-associated AF predominantly affects a younger demographic, frequently developing in patients in their 30s to 50s (54). Consequently, there is a high economic cost, driven by the direct upfront costs to health systems, and also by the loss of prime productivity.

Our study reveals a major deficit in recommendations for AF screening among RHD populations, highlighting significant gaps in current health policies and clinical guidelines. While the observed absence of formal recommendations likely reflects the lack of studies in this scenario, we believe that this is itself a notable finding warranting attention. We advocate for clinical steering committees and peak cardiovascular bodies to formally incorporate explicit recommendations for the early, opportunistic detection of AF within RHD guidelines. At minimum, this could serve to raise awareness and position AF screening in those with RHD as a research priority. Studies evaluating screening programmes must consider the expected downstream implications, including existing healthcare infrastructure, workforce capacity and pathway/access to AF treatment (55). The proposed screening modality and frequency should be tailored to the specific population and setting. For example, depending on factors such as rural or urban population, existing digital health infrastructure and remote transmission capability, there are options such as handheld devices capable of storing ECG tracings or continuous monitors with simultaneous remote transmission (56). Studies evaluating screening interventions should ideally quantify benefits and harms, establish cost-effectiveness and investigate the feasibility and acceptability among patients and healthcare workers (57,58,59).

In LMICs and remote settings, implementing screening protocols often requires the strategic empowerment of community health workers, nurses and rural generalists through upskilling to conduct initial screenings and manage ongoing care. A realistic framework must account for the scarcity of specialists, cost and availability of oral anticoagulation and geographic distances (60). To overcome these barriers, screening models may leverage mobile health technology and local capacity building. The integration of point-of-care diagnostics, nurse-led AF clinics, multidisciplinary care teams and telemedicine support can substantially improve AF-related outcomes (61).

### Strengths and limitations

Strengths of this review include the comprehensive nature of the document search and the inclusion of both AF-focussed and RHD-focussed guidelines across a range of geographic and health system contexts.

However, several limitations must be acknowledged. First, the review was limited to English-language policy documents and those accessible through publicly available databases or organisational websites, potentially excluding unpublished or locally adapted documents from low-resource settings. Secondly, as a narrative review, there is an inherent risk of subjectivity in interpretation and synthesis, although data extraction was standardised to reduce bias.

## Conclusions/Summary

This review highlights a clear gap in current guidelines and policies for AF screening recommendations in those with RHD. Although AF is a common complication of RHD, only one policy document was identified that provided specific recommendations for AF screening in patients with RHD. RHD is not adequately prioritised as a global priority for several reasons, including the perception that it mainly affects LMICs, and ongoing advocacy is needed. Further work is needed to inform and develop appropriate AF screening guidelines tailored to RHD populations in different settings.

## Data Availability

All relevant data are contained within the manuscript.
